# *Bletilla striata* polysaccharide cryogel scaffold for spatial control of foreign-body reaction

**DOI:** 10.1186/s13020-021-00526-y

**Published:** 2021-12-04

**Authors:** Jiaxi Chen, Huiqun Zhou, Daping Xie, Yiming Niu

**Affiliations:** State Key Laboratory of Quality Research in Chinese Medicine, Institute of Chinese Medical Sciences, University of Macau, Avenida da Universidade, Macau SAR, China

**Keywords:** *Bletilla striata* polysaccharide, Cryogel, Spatial regulation, Foreign-body reaction

## Abstract

**Background:**

Implantation of a biomaterial may induce the foreign-body reaction to the host tissue that determines the outcome of the integration and the biological performance of the implants. The foreign-body reaction can be modulated by control of the material properties of the implants.

**Methods:**

First, we synthesized methacrylated *Bletilla striata* Polysaccharide (BSP-MA) and constructed a series of open porous cryogels utilizing this material via the freezing-thawing treatment of solvent-precursors systems. Second, Pore size and modulus were measured to characterize the properties of BSP cryogels. Live/dead staining of cells and CCK-8 were performed to test the cytocompatibility of the scaffolds. In addition, the Real-Time qPCR experiments were carried for the tests. Finally, the BSP scaffolds were implanted subcutaneously to verify the foreign-body reaction between host tissue and materials.

**Results:**

Our data demonstrated that cryogels with different pore sizes and modulus can be fabricated by just adjusting the concentration. Besides, the cryogels showed well cytocompatibility in the in vitro experiments and exhibited upregulated expression levels of pro-inflammation-related genes (*Tnfa* and *Il1b*) with the increase of pore size. In vivo experiments further proved that with the increase of pore size, more immune cells infiltrated into the inner zone of materials. The foreign-body reaction and the distribution of immune-regulatory cells could be modulated by tuning the material microstructure.

**Conclusions:**

Collectively, our findings revealed *Bletilla striata* polysaccharide cryogel scaffold with different pore sizes can spatially control foreign-body reaction. The microstructure of cryogels could differentially guide the distribution of inflammatory cells, affect the formation of blood vessels and fibrous capsules, which eventually influence the material-tissue integration. This work demonstrates a practical strategy to regulate foreign body reaction and promote the performance of medical devices.

## Background

The rapid development of regenerative medicine has brought much promise to tissue maintenance, repair, and host defense [1–4]. As a prominent tool in regenerative medicine, tissue engineering has become an active field of scientific research for nearly three decades [5, 6]. The key to developing tissue engineering is the design of applicable and bioactive materials. With the development of materials science and tissue engineering, The design of biomaterial sophistication has also dramatically increased [7, 8]. In addition to the basic characters, such as biocompatibility, biodegradability, mechanical properties, biological activity has been placed in an important position considering of the further application in clinical [9, 10]. For instance, cell adhesion [11], vascularization [12], and biological recognition [13]. Therefore, polysaccharides, as a kind of biological polymers, have come to the stage [14].

Chinese medicines, as resources repository, have been widely applied in tissue repair and have been proven effective in the past years [15]. Among them, *Bletilla striata*, has been used as an astringent hemostatic medicinal for thousands of years [16, 17]. The medicinal part is generally regarded to be its pseudobulbs and it has the effects of restraining bleeding, reducing swelling, resolving mass and promoting tissue regeneration [16]. The effective component of it has been proved by modern pharmacology to be *Bletilla striata* polysaccharide (BSP) [18]. In general, Chinese medicine-derived polysaccharides are barely used in biomaterials. However, natural polysaccharides are demonstrated to be a potential biomaterial and have advanced interaction with tissue in diverse ways. EUP3, as the polysaccharide derived from *Eucommia ulmoides*, is demonstrated to have extraordinary affinity with platelet-derived growth factor-BB thus leading to a Growth Factor-affinitive scaffold [19]. Besides, the scaffolds from konjac glucomannan (KGM) polysaccharide were proved to modulate the activity of macrophages [20]. Beyond that, the *Bletilla striata* polysaccharide we mentioned above has already been designed into scaffolds and showed the capacity to promote angiogenesis [21]. Nevertheless, it’s obvious that there are many defects as well. First, it is difficult to extract pure and homogeneous polysaccharides from natural products, thus stable processes and appropriate characterization methods need to be established. Second, since nature-derived polysaccharides are not suitable to be used as biomaterials directly, the modification and design strategy of polysaccharides become more and more important for further applications.

Related researches have been done before and discovered that glucomannan can stimulate macrophages to produce pro-regenerative cytokines thus promoting angiogenesis and tissue repairing [22]. However, the BSP still has challenges to be a three-dimensional scaffold for tissue engineering. Firstly, the pore size of the scaffolds is an important factor for cell growth and tissue remodeling, which made us pay attention to cryogels. Cryogels are gel matrices in which polymerization occurs at subzero temperatures and solvent crystals are defrosted to form interconnected macropores network [23]. In addition, the foreign-body reaction (FBR) is the most common problem for biomaterials implantation, which is a result of the wound healing response altered by the presence of a foreign body [24–27]. In recent years, many studies reported that the structural and biophysical properties of the material are capable to affect the performance of host-biomaterial response including its composition, mechanical and material properties, surface topography and molecular landscape [28]. As a result, it’s feasible to modulate the FBR by regulating of material properties. In this study, we modified the BSP with a well-defined structure into a series of three-dimensional scaffolds with only one parameter changing: pore size and investigated the FBR and its potential application as tissue engineering scaffolds.

## Methods

### Materials

*Bletilla striata* (China Pharmaceutical Corporation-Canton, Guangzhou, China); *Bletilla striata* polysaccharide (BSP) was prepared following our laboratory established and reported protocol; Fetal bovine serum and DMEM medium were obtained from Life Technologies; Calcium AM/PI kit was purchased from shanghaiyisheng (China); CCK8 kit; GoTaq 2-Step RT-qPCR system was purchased from Promega; TRIzol Reagent was obtained from Sigma-Aldrich; Other chemicals and reagents were purchased from Sigma-Aldrich unless otherwise stated.

The primers of relevant genes were listed as follows:

Mouse beta-actin: Forward: 5′-GCTGGTCGTCGACAACGGCTC-3′.

Reverse: 5′-CAAACATGATCTGGGTCATCTTTTC-3′;

Mouse *Nos2*: Forward: 5′-CCAAGCCCTCACCTACTTCC-3′.

Reverse: 5′-CTCTGAGGGCTGACACAAGG-3′;

Mouse *Il1b*: Forward: 5′-GCAACTGTTCCTGAACTCAACT-3′.

Reverse: 5′-ATCTTTTGGGGTCCGTCAACT-3′;

Mouse *Tnfa*: Forward: 5′-ACGGCATGGATCTCAAAGAC-3′.

Reverse: 5′-AGATAGCAAATCGGCTGACG-3′;

Mouse *Mrc1*: Forward: 5′-GTGGTCCTCCTGATTGTGATAG-3′.

Reverse: 5′-CACTTGTTCCTGGACTCAGATTA-3′;

Mouse *Tgfb*: Forward: 5′-TGGAGCAACATGTGGAACTC-3′.

Reverse: 5′-TGCCGTACAACTCCAGTGAC-3′;

Mouse *Arg1*: Forward: 5′-CAGAAGAATGGAAGAGTCAG-3′.

Reverse: 5′-CAGATATGCAGGGAGTCACC-3′;

Mouse *Osm*: Forward: 5′-AACTCTTCCTCTCAGCTCCT-3′.

Reverse: 5′-TGTGTTCAGGTTTTGGAGGC-3′;

Mouse *Vegfa*: Forward: 5′-GTTCAGAGCGGAGAAAGCAT-3′.

Reverse: 5′-TCACATCTGCAAGTACGTTCG-3′;

### Synthesis of methacrylated BSP

Oxidation of BSP, the C6 primary hydroxyls of *Bletilla striata* polysaccharides are oxidized to C6 carboxylate groups by TEMPO/NaClO/NaClO_2_ oxidation system in sodium acetate buffer (0.2 M, pH 5.0). After stirring at 40 °C for 24 h, oxidation was quenched by adding excessive ethanol. The precipitate of oxidated BSP was collected by centrifugation. Then, oxidized products were dialyzed (MWCO: 3500) with milli-Q water, and lyophilized.

Preparation of methacrylated BSP: oxidized BSP was dissolved in a buffer solution (1% w/v, pH 6.5) of 50 mM 2-morpholinoethanesulfonic acid (MES). N-hydroxysuccinimide (NHS) and 1-ethyl-3-(3-dimethylaminopropyl)-carbodiimide hydrochloride (EDC) (molar ratio of NHS:EDC =1:2) were added to the solution to activate the carboxylic acid groups of the oxidized BSP. After activation for 5 min, AEMA (molar ratio of NHS: EDC: AEMA= 1:2:1) was added to the mixture and the reaction was maintained at room temperature for 24 h. The precipitate of methacrylated BSP was collected by centrifugation. Then, oxidized products were dialyzed (MWCO: 3500) with milli-Q water, and lyophilized.

### Characterization of methacrylated BSP

Characterization of oxidized BSP: oxidized BSP was dissolved in deuterated dimethylsulfoxide (DMSO-d6), and the ^13^ C NMR spectra of these glucomannan/DMSO solutions were recorded. Carboxylate content of oxidized BSP was determined by the potentimetric titration method. 0.1 M HCl was added to methacrylated BSP solution and set pH value in the range of 2.5–3.0, then record HCl volume. 0.1 M NaOH solution was added up to pH11 and record NaOH volume.

Characterization of methacrylated BSP: methacrylated BSP was characterized by ^1^H-NMR analysis and the efficiency of BSP methacrylation was determined from ^1^H-NMR spectra based on the ratio of the integrals for the internal standard protons to the methylene protons of methacrylate.

The FTIR spectra of lyophilized pure BSP and BSP-MA were obtained on KBr pellet performed on a FTIR spectrophotometer (MAGNA IR560, Nicolet). All spectra were recorded with the resolution of 4 cm^−1^ in the range 400–4000 cm^−1^.

### Methacrylated BSP scaffolds preparation

GM scaffolds preparation: methacrylated BSP solution (2%–10%) was synthesized using deionized water as a solvent. Then add tetramethylethylenediamine (TEMED) [0.5% (wt/vol)] and ammonium persulfate (APS) [0.25% (wt/vol)] to the methacrylated BSP solution which was precooled to 4 °C to decrease the rate of polymerization. After a complete incubation in −20 °C refrigerator for one night, the cryogels were put at room temperature to remove ice crystals and washed with milli-Q water.

### Pore size and rheology measurement of methacrylated BSP scaffolds

GM scaffolds were stained with fluorescent dyes (FITC). A solution of FITC, 1 mM in 20 mM Na-carbonate buffer (pH 9.4) was applied to the stained scaffold for 24 h and thoroughly washed with buffer and water. The stained cryogels were sectioned into slices. Samples were examined by confocal laser scannin microscopy (CLSM) (Leica TCS SP8), using a 20× objective and excitation and emission wavelengths 488 and 530 nm. ImageJ software (http://rsb.info.nih.gov/ij/) was used to analyze images to obtain the pore size and pore size distribution.

Flow and deformation of materials in response to applied force can be studied by rheology. Elastic modulus and elastic nature of the material is defined as storage modulus (G′). The dissipation (viscous) of the flow is represented by loss modulus (G″). The visco-elasticity behavior or phase angle is the difference between the storage and loss modulus. The cryogels used in experiments were 1.5 cm in diameter and 2 mm in thickness cylindrical shape. Amplitude sweep (strain sweep) test was applied from 0.01 to 100% at the constant frequency of 1 Hz to determine linear viscoelastic region. Then, frequency sweep measurement was performed from 0.01 to 100 Hz at a controlled strain of 0.2% to investigate the modulus change related to the oscillatory frequency.

### Cell culture

RAW 264.7, a murine monocyte/macrophage cell line, and human umbilical vein endothelial cells (HUVECs) were purchased from the ATCC (American Type Culture Collection). Cells were cultured in DMEM high glucose medium and RPMI-1640 medium supplemented with 10% FBS and 1% penicillin/streptomycin under 5% CO_2_ at 37 °C. Cells were passaged after reaching 80% confluence.

### Live/dead staining of cells in scaffolds

RAW 264.7 macrophages and HUVECs were washed by PBS. After cell counting, the cells were centrifuged and re-suspended in culture medium solution at a final concentration of 5 × 10^6^ cells per milliliter. 3D scaffolds were sterilized in 75% ethanol for one night. Next, the cell solution was added to the 3D scaffolds and cells can be absorbed into the 3D scaffolds. The cell-laden cryogels were then placed in the atmosphere of 37 °C with 5% CO_2_ for 6 h to allow cells attachment inside the scaffold.

A live/dead assay was performed to test cell viability in cryogels. Scaffolds loaded with cells in triplicate were incubated with the mixture dye solution containing 1µL of Calcein-AM and 0.5µL propidium iodide (PI) in 1 mL of PBS. After 30 min incubation, the cryogels were rinsed with PBS and cells were imaged by confocal laser scanning microscopy (CLSM) (Leica TCS SP8). Green fluorescence represents live cells and red fluorescence was related to dead cells.

### Cell proliferation assay in scaffolds

RAW 264.7 macrophages and HUVECs were seeded in scaffolds as previously mentioned. Scaffolds were placed in a 96-well plate at the density of 2 × 10^5^ cells/scaffold. 6 h after seeding, the cell-laden scaffolds were rinsed with PBS and transferred to another new well to remove the unattached cells. To evaluate cell proliferation, at day 1 and 3, the culture medium was replaced with the cell counting kit-8 (CCK-8) working solution and incubated at 37 °C for 3 h. The CCK-8 solution was collected and the absorbance value was measured with the multi-plate reader at wavelength of 450 nm.

### Cell infiltration and distribution in scaffolds

RAW 264.7 macrophages were seeded in scaffolds as previously mentioned. Calcein-AM solution was added to the sample. After 30 min incubation at 37 °C, the cell-laden cryogels were observed by CLSM. All images were generated by optical sectioning in the z-direction. Optical sections each of 10 μm were taken to produce a 250 μm z-stack for image processing.

### Real-Time qPCR

RAW 264.7 macrophages were seeded in scaffolds at a seeding density of 5 × 10^4^ cells/scaffold. Cell-laden scaffolds were rinsed with PBS and transferred to another new well to remove the unattached cells and 1.5 ml of new culture medium was added. Then, samples were incubated in CO_2_ incubator for 24 h.

RNA was isolated by kit. RNA was reverse-transcripted into cDNA. Quantitative real-time PCR (q-PCR) measurements were performed using a SYBR Green RT-PCR kit. Marker genes including *Tnfa*, *Il1b*, *Nos*, *Mrc1*, *Tgfb*, *Arg1*, *Vegfa* and *Osm* were selected for analysis with the primer sequences using the 2 −ΔΔct relative quantification method.

### Implantation of cryogel scaffolds

Male C57BL/6J mice (6–8 weeks) were used with cryogel scaffolds subcutaneous implanted in the back for assessing the host response and the biocompatibility of cryogel scaffolds. All procedures were approved by the Animal Ethics Committee, University of Macau. We divided 18 mice into three groups and treated them with hydrogel and BA2 and BA8 respectively embedded. The mice in each group were divided into 2 days and 14 days.

Before surgery, the mice were anesthetized with an intraperitoneal injection of sodium pentobarbital (70 mg/kg) and then the dorsal hair was shaved. After the sterilization of skin with 75% ethanol, two independent incisions were made on the back, and the scaffolds were embedded in and then the wound was sewn up.

### Histology analysis

After two days and 2 weeks of housing, the mice were sacrificed and the implants along with the 2 cm × 2 cm skin tissue samples were collected and immediately fixed in 4 vol% formalin and dehydrated by gradient ethanol before embedding in paraffin wax. These samples were cross-sectioned into 6 μm for histological analysis. The sections were deparaffinized and rehydrated for Hematoxylin–eosin (H&E), Masson’s trichrome staining (M&T). Besides, the deparaffinized and rehydrated sections were blocked and stained by anti-VEGF, anti-CD31 and anti-CD86 for immunohistochemistry. The images were recorded by a light microscope (BX51; Olympus). ImageJ software was utilized to quantify the fibrous capsule thickness, immunohistochemistry staining (with the assistant of IHC Toolbox plugin), and obtain the cell coordinate datasets followed by calculating the minimum cell distance in R language.

### Statistical analysis

Statistical differences among samples were studied through t-test or the one-way analysis of variance (ANOVA) with Tukey’s multiple comparisons test. The data presented as the mean ± standard deviation was obtained based on at least three independent replicates. Significance was set to p < 0.05. (*p < 0.05, **p < 0.01, ***p < 0.001, ****p < 0.0001).

## Results

### Synthesis and characterization of glucomannan derivates

**Fig. 1:**
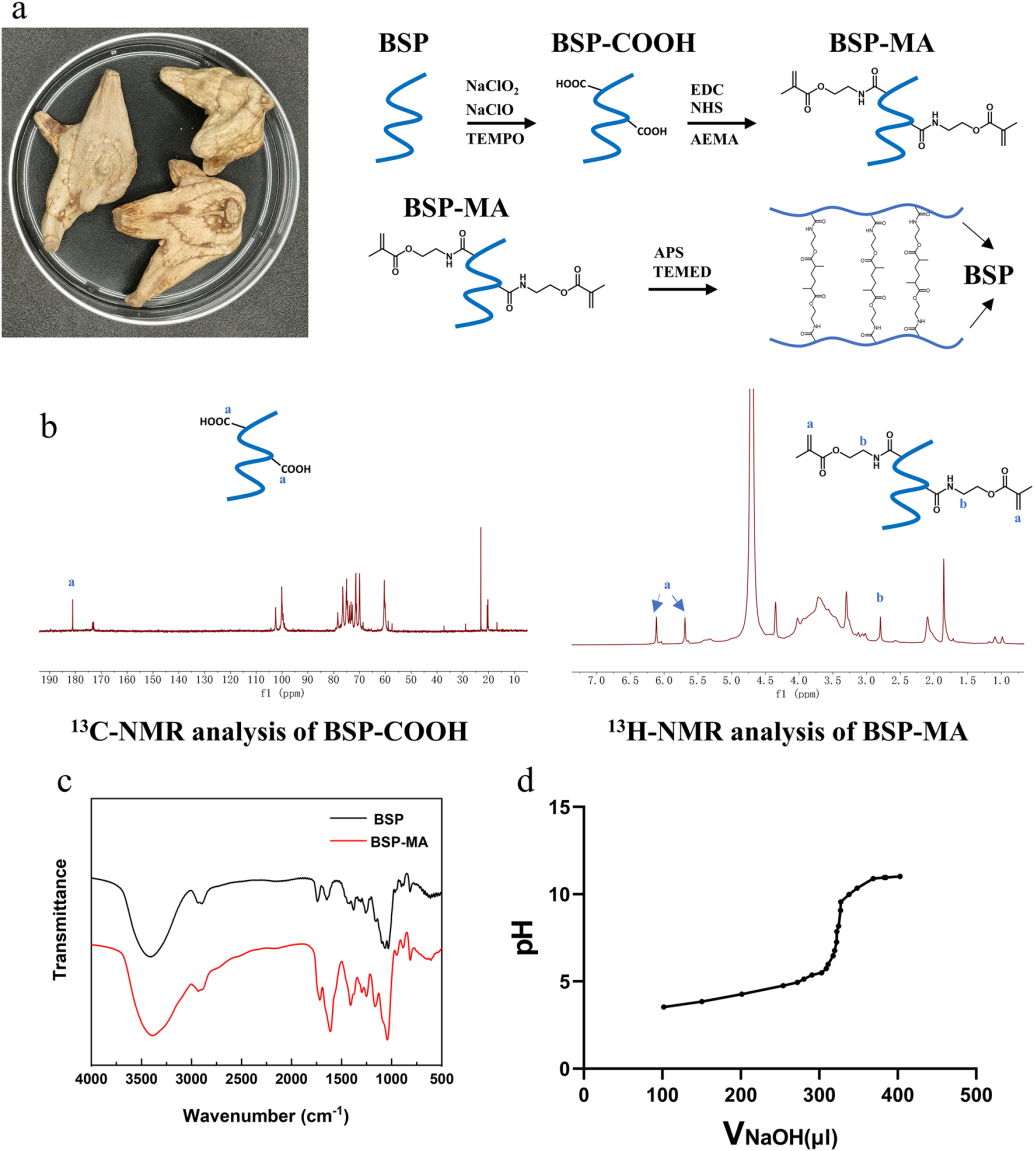
Preparation and characterization of methacrylated BSP. **a** The Chinese medicine *Bletilla striata* and Schematic illustration of methacrylated BSP synthesis procedures and the polymerization under the TEMED/APS initiation system. **b** ^13^ C-NMR and ^1^H-NMR spectrum analysis of oxidized BSP and methacrylated BSP. **c** The FT-IR of BSP and BSP-MA. **d** Potentiometric titration for degree of substitution

There is no report of methacrylated on BSP up to now. Therefore, we first propose a modification method of BSP to MA. C6-OH groups of BSP are oxidized to C6 carboxylate groups by TEMPO/NaClO/NaClO2 oxidation system (Fig. 1a) [29]. The ^13^ C-NMR spectrum with the peak at 180ppm indicated that C6 primary hydroxyls were successfully oxidized to carboxylate groups (Fig. 1b). Determined by potentiometric titration method, the carboxylate content of C6-OH was 2.12 mmol/g and the oxidation ratio of C6-OH was 37.06%. (Fig. 1d) Then these carboxylate groups are linked with AEMA to introduce a carbon-carbon double bond, which can be crosslinked via the chemical method calculated, TEMED/APS initiation system based on an autocatalytic reaction (Fig. 1a). At last, BSP was successfully methacrylated.

The ^1^H-NMR spectra of methacrylated BSP exhibit peaks of vinyl methylene and methylene protons that were newly formed by the reaction with AEMA, which are located at δ 6.2, 5.7 and 2.9 respectively (Fig. 1b). The FIIR also confirms the successful synthesis of methacrylated BSP (Fig. 1c): the single peak of 1600 cm^−1^ presented the stretching frequency of C=C in the alkene. These results demonstrated that BSP was successfully methacrylated. To calculate the efficiency of BSP methacrylation, the ^1^H-NMR spectra were recorded using tetramethylsilane (TMS) as an internal standard. The efficiency of BSP methacrylation was calculated to be 20.66% based on the ratio of the integrals for the internal standard protons to the methylene protons of methacrylate (Fig. 1b).

### Fabrication and characterization of methacrylated BSP scaffolds

**Fig. 2:**
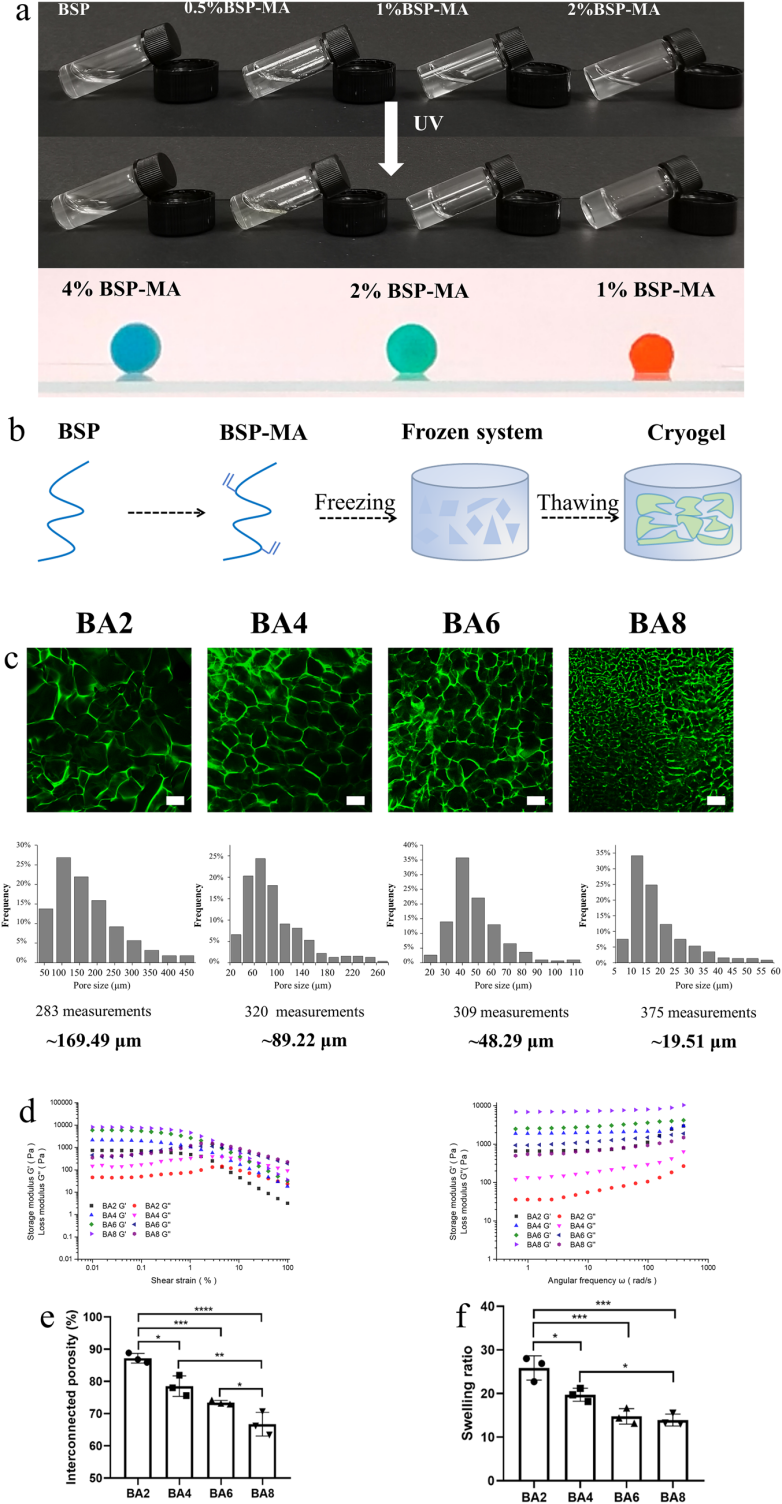
Fabrication and characterization of BSP-based cryogels. **a** Fabrication of hydrogels and cryogels with different concentrations **b** Schematic depiction of cryogels preparation. **c** Confocal laser scanning fluorescence microscopy (CLSM) analysis of BA2–BA8 cryogels [varying concentration of the precursor solution: 2%, 4%, 6%, 8%, (w/v)%], average pore diameters and the pore size distributions. Scale bars: 100 μm, 50 μm, 20 μm, 20 μm. **d** Rheological analysis of BA2–BA8 cryogels. Storage modulus (G′) and loss modulus (G′′) of BA2–BA8 cryogels on strain sweep measured at 1 Hz of frequency (left), frequency sweep measured at 0.2 % of strain (right). **e** Interconnected porosity (n = 3) and **f** swelling ratio (n = 3) of BA2–BA8 cryogels. Statistical analysis: Error bars represent standard error (n = 3). One-way analysis of variance (ANOVA) with Tukey’s multiple comparisons test, *p < 0.05, **p < 0.01, ***p < 0.001, ****p < 0.0001

The process of methacrylation makes the BSP crosslinking to form hydrogel through simply ultraviolet (UV) radiation. The BSP still displays liquid status after 15s UV radiation, however, the BSP-MA with different concentrations 0.5%, 1% and 2% were tested to show the different obviously capacities of gelation after the same radiation time. (Fig. 2a) The 2% BSP-MA presented to change the liquid state to solid state demonstrating the capacity of BSP-MA gelation. However, the pore size in hydrogel is much smaller than cells which is not suitable for cell infiltration and angiogenesis.

Cryogels are gel matrices in which polymerization occurs at subzero temperatures and solvent crystals are defrosted to form an interconnected macroporous network [30]. BSP cryogels were prepared by freezing and thawing (Fig. 2b). The freezing step causes the ice crystals to form and occupy space, while the subsequent thawing step causes the ice crystals to melt, thus forming large interconnected pores. It showed the macroscopical performance of cryogel BSP-MA, the three colors represent three different concentrations of BSA-MA (Fig. 2a). It’s obvious that red one with lowest BSP-MA concentration performs a sort of collapse. To explore the concentration effect on material properties, we prepared cryogels with four concentrations of BSP-MA and named BA2 (2%) BA4 (4%), BA6 (6%) and BA8 (8%) (Fig. 2c). The concentration can affect the mechanical properties by affecting the pore size [31]. As a result, the cryogels were stained with FITC and pore size and frequency were measured. It’s shown that BSP cryogels contained tunable pore sizes of 169.49, 89.22, 48.29 and 19.51 μm (Fig. 2c), The pore size gradually decreases with the increase of concentration. The BA2 and BA4 presented the largest pore size.

Next, to observe the inner network and analyze the structure and distribution of the pore in each cryogel group, we measured the swelling ratios and the interconnected porosity. We found that through adjusting the concentration of the initiator and the precursor BSP-MA solution, we developed cryogels with tunable pore size. With the increase of concentration of the precursor BSP-MA solution, porosity was decreased, and some of them were even about 90% (Fig. 2e), while the swelling ratios of cryogels declined from 25 to 13 (Fig. 2f).

Rheological properties were also investigated to characterize the cryogels to confirm the dimensional stability of the inner work [32]. Rheology test demonstrated that cryogels of different concentrations exhibited diverse stiffness at a range from 709.1576 (storage modulus, Pa) to 7803.308 (storage modulus, Pa) (Fig. 2d). As a result, the modulus regulation can be achieved by adjusting pore size, which is a key factor for biomaterial application.

### Cytocompatibility analysis of the cryogel-based scaffolds

**Fig. 3:**
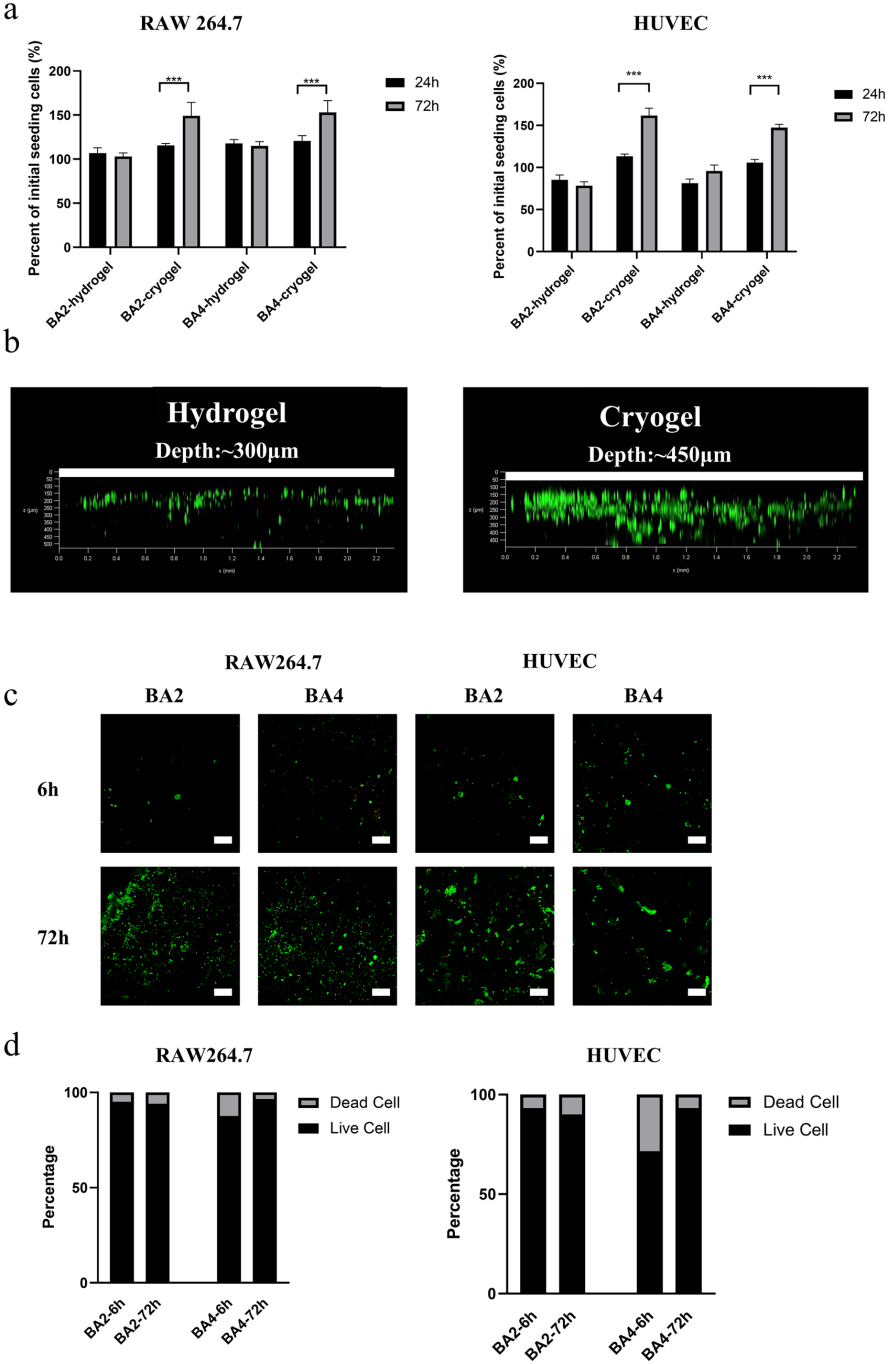
Cytocompatibility analysis of cell-laden cryogel: **a** RAW 264.7 cell and HUVECs viability on BA2 hydrogels and cryogels and BA4 hydrogels and cryogels after 24 and 72 h culture. **b** Infiltration and distribution images of RAW 264.7 cells seeded on hydrogel and cryogel for 24 h. **c**, **d** Representative images and quantitative analysis of live/dead staining of RAW 264.7 macrophages and HUVECs in BA2 and BA4 cryogels after 6 and 72 h of culture. Scale bar: 200 μm. Statistical analysis: Error bars represent standard error (n = 3). T-test, *** p < 0.001

To evaluate the cytocompatibility of the cryogels as a biomaterial, we chose BA2 and BA4 cryogels to culture cells due to their relatively large pore size, which is proved to be suitable for cell infiltration and tissue growth. As a control, the same concentration of hydrogel was also prepared. As the previous work showed that BSP could regulate macrophages and promote angiogenesis, RAW 264.7 cells and HUVECs were chosen to seed in BA2 and BA4 to evaluate cell distribution and viability. After 6 and 72 h of culture, RAW 264.7 cells and HUVECs proliferated well in the BA2 and BA4 scaffolds without any significant difference (Fig. 3a). Compared with cells seeded on hydrogels, OD values of cells increased by around 1.5 times after culturing for 3 days (Fig. 3a).

To further compare the cell infiltrating between hydrogel and cryogel, we use live/dead staining. RAW 264.7 cells could infiltrate even up to 450 μm depth in cryogel while the hydrogel can only infiltrate up to 200 μm after culturing for 1 day, indicating the construction of interconnected macropores (Fig. 3b). Besides, both BA2 and BA4 cells had high survival rates at 6 and 72 h in RAW 264.7 cells and HUVACs. (Fig. 3c) To quantification the ratio of live/dead cells, we used Image J to analyze the fluorescence. (Fig. 3d) For HUVACs, the BA2 showed 93.3% live ratio in 6 h and 90.1% in 72 h, BA4 showed 71.5% in 6 h and 93.3% in 72 h. For RAW 264.7 cells, the BA2 showed 95.2% live ratio in 6 h and 94.1% in 72 h, BA4 showed 87.6% in 6 h and 96.5% in 72 h. These results demonstrated that BA2 and BA4 cryogels were excellent substrates for cell growth.

### GM biomaterial-based mechanical modulation of macrophages

The above work proves that by only adjusting the concentration, the cryogel can be adjusted to have different mechanical properties, such as pore size, modulus, etc. Besides, cytocompatibility experiments have proved that cryogels are suitable for cells growth. Therefore, in order to investigate the FBR, the expression levels of macrophage-related genes were measured by RT-qPCR performing.

**Fig. 4:**
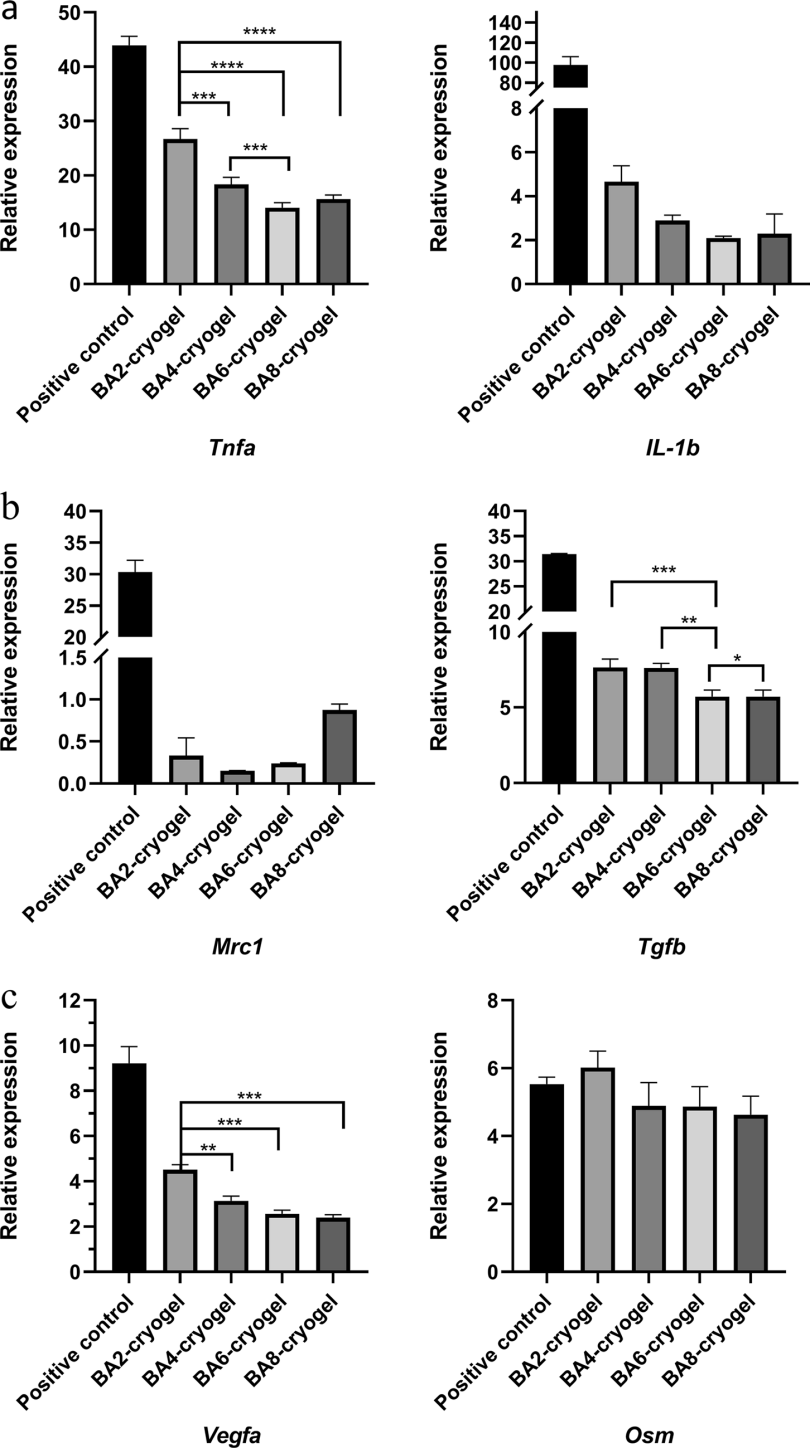
Gene expression of RAW 264.7 macrophages response to BSP based cryogels with varying stiffness. qPCR analysis of gene: *Tnfa* (**a**), *Il1b* (**a**), *Mrc1* (**b**), *Tgfb* (**b**), *Vegfa* (**c**) and *Osm* (**c**) expression of macrophages in response to BA2–BA8 cryogels. Statistical analysis: Error bars represent standard error (n = 3). One-way analysis of variance (ANOVA) with Tukey’s multiple comparisons test, *p < 0.05, **p < 0.01, ***p < 0.001, ****p < 0.0001

We evaluated the pro-inflammatory related genes (*Tnfa*, *Il1b*),  anti-inflammatory related genes (*Mrc1*, *Tgfb*) and repair related genes (*Vegfa *and *Osm*) (Fig. 4). in RAW264.7 after 24 h culture. It’s exhibited increased expression levels of pro-inflammation-related genes including *Tnfa* and *Il1b*. (Fig. 4a) Specifically, with the increase of pore size, the up regulation of *Tnfa* and *Il1b* increases obviously from BA8 to BA2, and BA6 has a similarly low level with BA8. It’s common that the pro-inflammatory cytokines increase because of the cryogels intervention. However, it is worth noting that the pro-inflammatory cytokines gradually decreased as the pore size became smaller, which preliminarily proved that regulating the pore size can modulate inflammatory reactions. The *Tgfb*as a pleiotropic cytokine was upregulated at the comparable level regardless of the pore size. (Fig. 4b) Meanwhile, anti-inflammation-related genes such as *Mrc1* was down regulated (Fig. 4b). In addition, the expression of angiogenesis-associated gene *Vegfa* and osteogenesis-related gene *Osm* genes were increased at the same time (Fig. 4c). Therefore, we found that our materials can promote the M1 polarization of macrophage, and the degree of pro-inflammation declined gradually with the decreased of the pore size of cryogels.

### In vivo foreign-body reaction modulated by pore size

To evaluate the biocompatibility and foreign-body reaction of the materials in vivo, we subcutaneously implanted BA8 and BA2 cryogels and BSP hydrogels for 2 days and 14 days. It’s revealed that the materials with different pore sizes could regulate the host response from histological staining results.

**Fig. 5:**
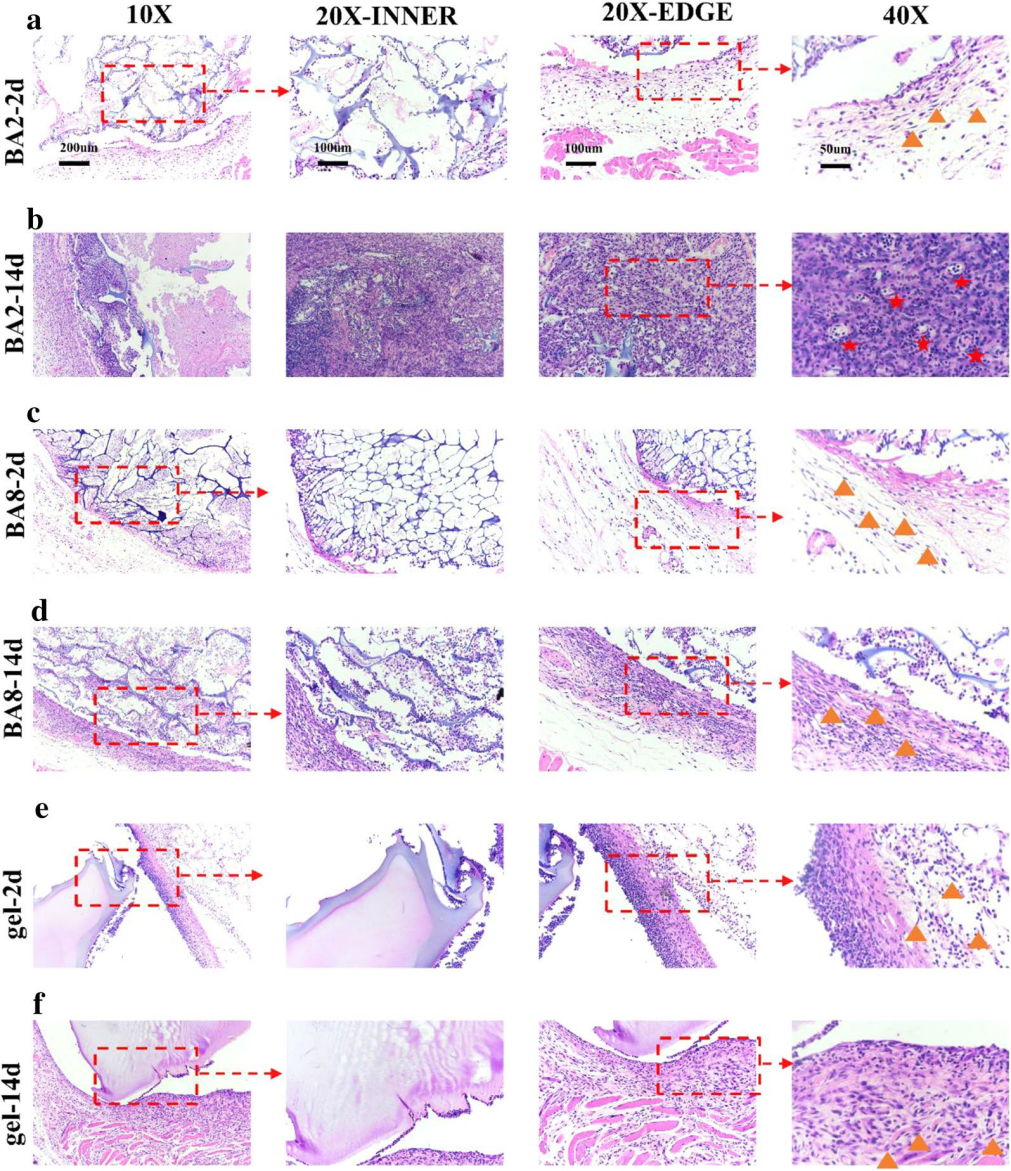
Immune cell infiltration in materials. H&E staining images of BA2 subcutaneously implanted in mice for 2 days (**a**) and 14 days (**b**), and BA8 implanted for 2 days (**c**) and 14 days (**d**), hydrogel implanted for 2 days (**e**) and 14 days (**f**). (Yellow triangle: location of fibroblasts; Red star, new blood vessels)

Firstly, the distribution of immune cells can be regulated by the pore size of materials. It’s shown that the materials still have similar morphology as it was measured in vitro after implantation. BA2 has interconnected macropores and BA8 has interconnected smaller pores, while hydrogel has no obvious pores as a whole material (Fig. 5).

**Fig. 6:**
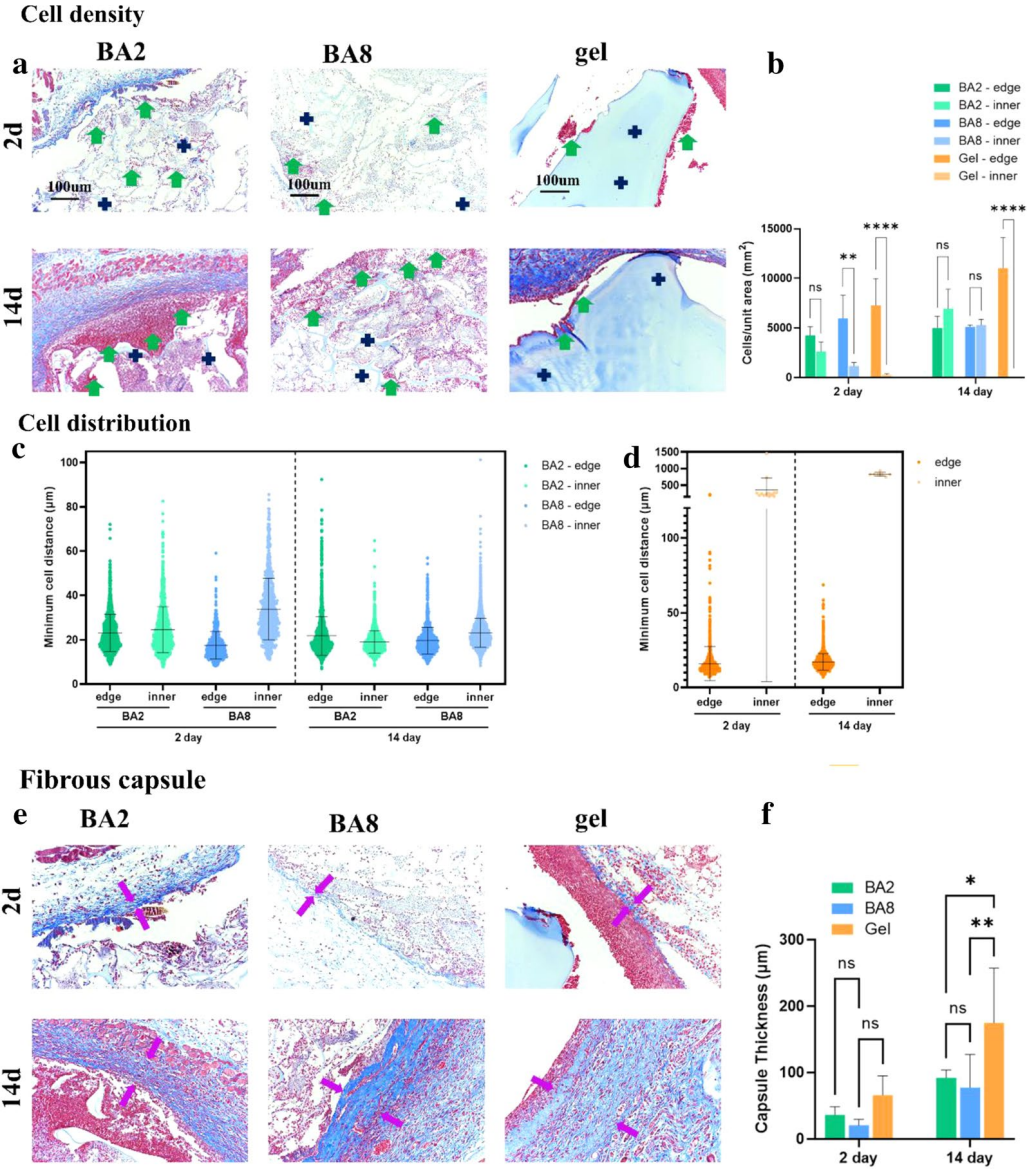
The cell distribution and the formation of fibrous capsule in tissue surrounding the BA2, BA8 and hydrogel. **a** The M&T staining of BA2, BA8 andhydrogel after 2 days and 14 days implantation. **b** shows the quantification of the immune cells density. **c**, **d** shows the quantification of cell distribution. **e** M&T staining of fibrous capsule and the **f** thickness quantification. (Green arrows, infiltrating immune cells; Blue crosses, materials; Purple arrows, thickness and location of fibrous capsule)

**Fig. 7:**
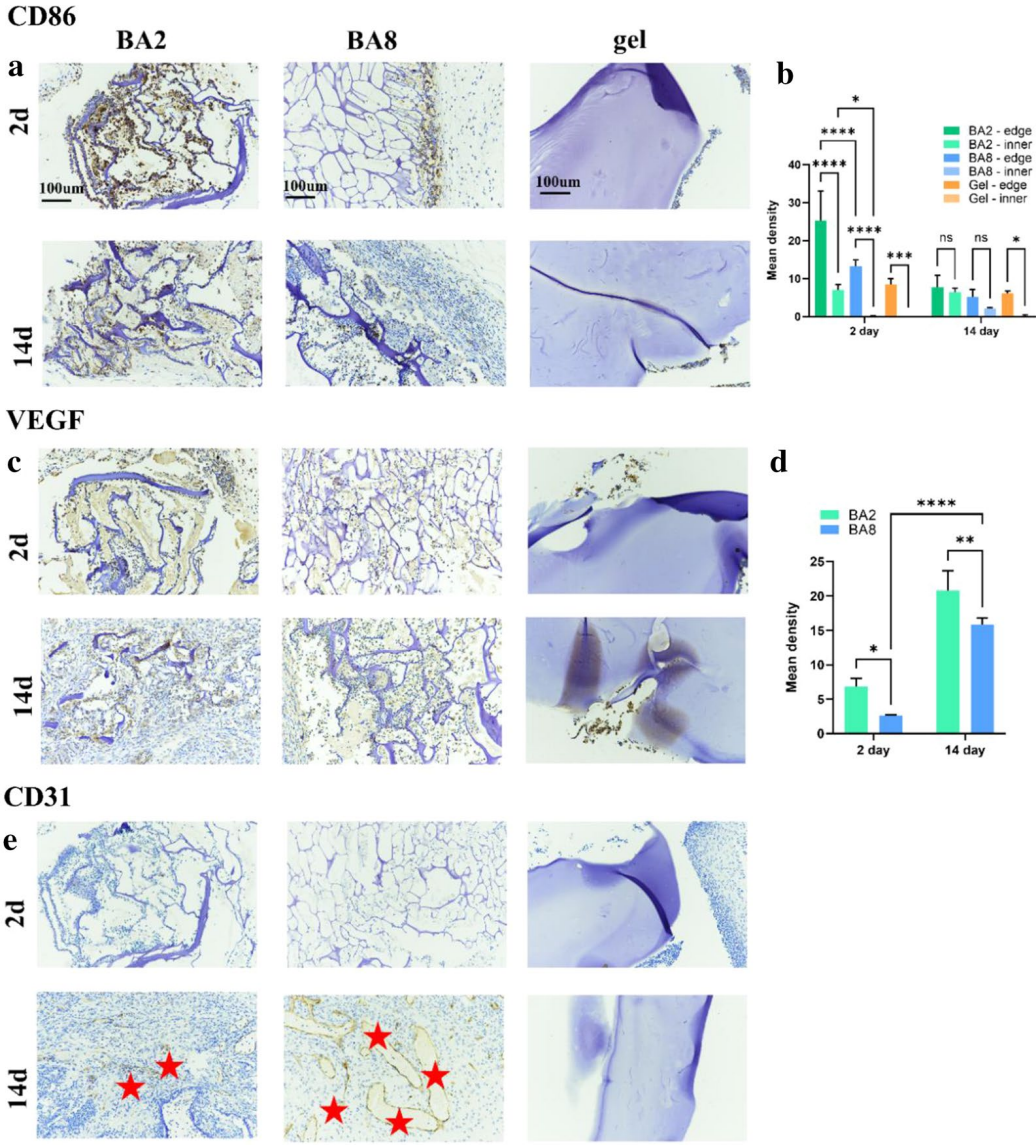
Inflammatory marker staining and qualification analysis of BA2, BA8 and hydrogel. **a** Inflammatory staining and **b** qualification analysis of CD86 **c** inflammatory staining of VEGF and **d** qualification analysis, **e** inflammatory staining of CD31 (Red stars, new blood vessels)

We use Hematoxylin-eosin (H&E) staining and Masson’s trichrome (M&T) to present the host response and distribution of immune cells in the implantation site []. After 2 days, for BA2, the materials with the largest pore size, the immune cells were distributed in both inner and edge of materials (Figs. 5, 6). The quantitative data (Fig. 6c) which measured the minimal cell distance and gave an visualized map of the immune cells distribution further demonstrated the comparable amount of inner and edge of the BA2. BA8, with the smaller pore size, immune cells infiltrated both inner and edge as well, but the immune cells concentrated more at the edge of the BA8 (Fig. 6c). For hydrogel, with no macropores, immune cells only exist at the edge of the material, with little inner presence (Fig. 6d). After 14 days, the immune cells in both BA2 and BA8 distributed more evenly compared with them after 2 days. However, hydrogel group still showed the immune cells concentrating at the edge of materials. The above results indicated that the pore size can mediate the FBR by regulating the distribution of immune cells. At the initial stage of material implantation, the large the pore size is, the easier it is for immune cells to infiltrate into the inner of the materials. With the decrease of the pore size, the immune cells are distributed to the edge of the materials. The quantitative data which measured the ratio of cell and unit area showed the cell density of inner and edge materials demonstrated the distribution trend with the pore size (Fig. 6b). Immunohistochemical staining of CD86 was also performed to identify the location of macrophages in the cryogels (Fig. 7a). Macrophages, as myeloid immune cells which play the role of pioneer of the FBR, can ingest and degrade dead cells and foreign materials in addition to orchestrating inflammatory processes [33].The distribution of macrophages is similar to that of overall immune cells. Notably, BA2 material showed a higher density of macrophages than BA8, which was consistent with the levels of inflammatory cytokines in vitro.

Secondly, the formation of fibrous capsules can be modulated by the pore size of materials [34]. In tissues exposed to the system’s immune system, lymphocytes and fibroblasts develop fibrous capsules to fight against biological materials. We evaluate the thickness of the fibrous capsule as an indicator of the FBR. M&T staining (Fig. 6) exhibited that the fibrous capsule of BA8 is a litter bit thinner than it of BA2, but the quantification showed no significant difference. Besides, hydrogel has the thickest fibrous capsules (Fig. 6e, f). This may be due to the macropore of BA2 and BA8, which leads to more M1 macrophages entering the materials and ingesting the materials. While the macrophages of hydrogel concentrating at the edge turned into M2 macrophages and secreted the anti-inflammation cytokines, including Tgfβ, which promoted the collagen deposition and the formation of fibrous capsules [35].

**Fig. 8:**
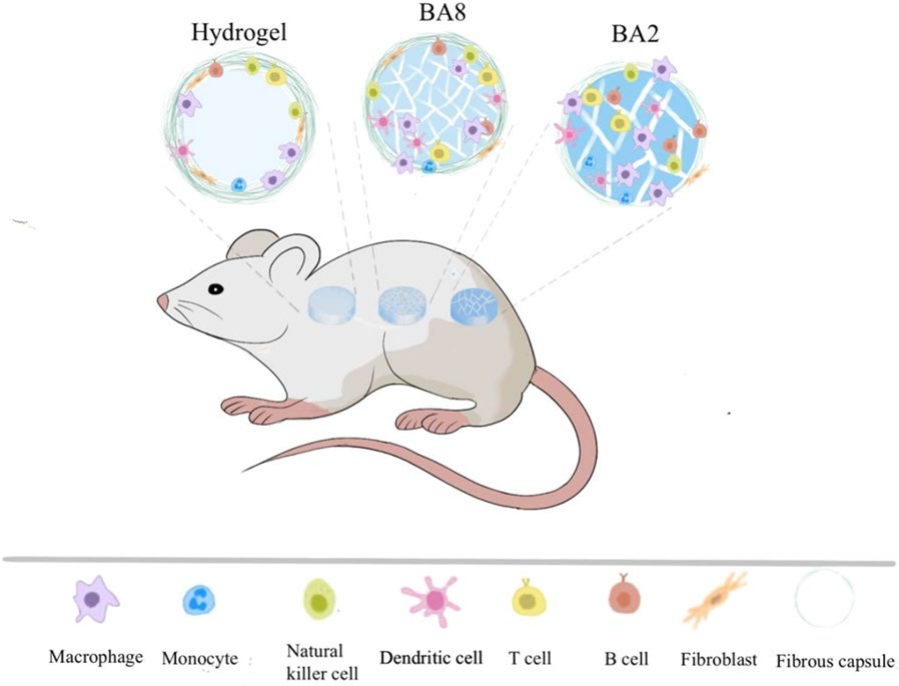
The schematic diagram for spatial control of foreign-body reaction by modulation the pore size. The immune cells diversely distributed in the materials with different pore size and the different levels of fibrous capsule formation

For implantation of biomaterials, tissue compatibility is also a very important consideration [36, 37]. The pore size can affect the integration of materials and tissues. Immunohistochemical staining of VEGF and CD31 was measured to evaluate angiogenesis (Fig. 7c–e). It’s found that the expression level of VEGF in vivo is consistent with that measured in vitro. BA2 expressed the most VEGF in contrast with BA8 at both 2 and 14 days. Besides, the angiogenesis was exhibited at 14 days which indicated the formation of tissue in BA2 and BA8 (Fig. 7e). In addition, BA8 has better tissue compatibility (Fig. 7), BA2, with large pore size, allowed macrophages to infiltrate to inner material thus leading to more severe inflammation, which is not conducive to blood vessel growth and tissue integration. The schematic diagram illustrated of the probable immune cells infiltration (Fig. 8).

## Discussion

In this study, we have developed a new, natural polysaccharide-based cryogel scaffold, which is effective to regulate host response. Notably, this polysaccharide is a Chinese medicine herb-derived glucomannan, exhibiting high biocompatibility and stability as a 3D scaffold.

Along with the repaid progress of tissue engineering normal biomaterial with biological functions and tunable physical properties are in high demand [38]. On the one hand, natural polysaccharides from the ocean have been widely developed [39–41]. On the other hand, polymers from Chinese medicine herbs, despite the evidence of bioactivity, still have much room for development, key obstacles include unclear composition and difficulties for material fabrication [42–44]. Here, for the first time, we made macroporous BSP gels suitable for cell culture just by freezing and thawing without adding any pore-forming agents.

The in vitro and in vivo data verified our hypothesis, which is the pore size can induce different levels of host response. Firstly, the immunohistochemistry staining of CD86 and VEGF results are agreed with the in vitro results of RT-qPCR which presented the consistency between the in vitro and in vivo data. Secondly, perhaps the most remarkable finding is that the material we successfully made can differentially guide to the distribution of immune cells, blood vessels, fibrous capsules. for example, Figs. 5, 6 and 7 showed the distribution and amount of macrophages and other immune cells are associated with the pore size of materials. BA2, with largest pore size, has more macrophages infiltrated and showed more internal distribution, which is also consistent with the *in vitro* data: the upregulation of pro-inflammatory genes. In addition, many studies have proved that foreign-body reaction can be modulated by various material properties such as porosity [45, 46].

These exciting findings suggest the future works for the development of BSP cryogel system. First, much work remains to do on identifying the specific types and subpopulations of the immune cells into the scaffolds at different time points, also interestingly, how these differential immune cells profiles lead to tissue remodeling and repair. Second, as a proof of concept study, we implant the gels in healthy mice, we will evaluate the general potential in specific disease models in future. We believe our exploration will open up a new avenue for the development of Chinese medicine resources for broad applications.

## Conclusions

Taking together, our works revealed that *Bletilla striata* polysaccharide cryogel scaffolds with different pore sizes can spatially control foreign-body reaction. The microstructure of cryogels could differentially guide the distribution of inflammatory cells, affect the formation of blood vessels and fibrous capsules, which eventually influence the material-tissue integration. This work demonstrates a practical strategy to regulate foreign-body reaction and promote the performance of medical devices.

## Data Availability

Not applicable.

## Abbreviations

FBR: Foreign-body Reaction
BSP: *Bletilla striata* polysaccharide
GM: Glucomannan
MES: 2-Morpholinoethanesulfonic acid
NHS: N-hydroxysuccinimide
EDC: 1-ethyl-3-(3-dimethylaminopropyl)-carbodiimide hydrochloride
DMSO: Dimethylsulfoxide
TEMED: Tetramethylethylenediamine.
APS: Ammonium persulfate
CLSM: Confocal laser scanning microscopy
HUVECs: Human umbilical vein endothelial cells
ATCC: American Type Culture Collection
CCK-8: Cell counting kit-8
q-PCR: Quantitative real time PCR
H&E: Hematoxylin–eosin
M&T: Masson’s trichrome
